# Predicting Suicide in Counties: Creating a Quantitative Measure of Suicide Risk

**DOI:** 10.3390/ijerph19138173

**Published:** 2022-07-04

**Authors:** Kate Mobley, Gita Taasoobshirazi

**Affiliations:** School of Data Science and Analytics, Kennesaw State University, 3391 Town Point Dr. NW, Suite 2400, MD 9104, Kennesaw, GA 30144, USA; gtaasoob@kennesaw.edu

**Keywords:** suicide, suicide prevention, quantitative analysis, public health, mental health

## Abstract

Rising rates of suicide over the past two decades have increased the need for wide-ranging suicide prevention efforts. One approach is to target high-risk groups, which requires the identification of the characteristics of these population sub-groups. This suicidology study was conducted using large-scale, secondary data to answer the question: using the research on suicide, are there variables studied at the community level that are linked to suicide and are measurable using quantitative, demographic data that are already collected and updated? Data on deaths from suicide in U.S. counties for the years 2000, 2005, 2010 and 2015 were analyzed using multiple regression, longitudinal regression, and cluster analysis. Results indicated that the suicide rate in a county can be predicted by measuring the financial stability of the residents, the quality of mental health in the county, and the economic opportunity in the county. The results are further analyzed using two sociological theories, Social Strain Theory and the Theory of Anomie, and two psychological theories, the Shame Model and the Interpersonal Theory of Suicide.

## 1. Introduction

In the United States, the rate of death by suicide rose from 10.4 deaths per 100,000 people in 2000 to 14.21 deaths per 100,000 people in 2018, the most recent year for which accurate data are available. Nationwide, during these 18 years, there was a 35.6% increase in the rate of completed suicides. The rate of death by suicide in children and adolescents aged 5 to 24 increased by 45%. Among children and adolescents aged 10 to 24, suicides rose from being the third most common cause of death to the second most common cause of death [1]. Between 2000 and 2010, the rate of death by suicide among children and adolescents increased just 2.3%, but between 2011 and 2018 there was a 41.7% increase [1]. Most recently, suicide risk factors for several populations have been exasperated by the current economic, social, mental, and physical distress caused by the COVID-19 pandemic.

While mental health resources are crucial to preventing suicide in individuals, in many cases there is not enough time to connect someone to mental health resources. Research has found that the time between making a plan and completing suicide often lasts ten minutes or less [2]. The time-sensitive nature of these crises means that they require immediate response, but also that proactive planning and prevention efforts are important not only in lowering the chances of a suicide crisis occurring, but also improving response methods when they do occur. Suicide prevention methods fall into three categories: Those which target the entire population, those targeting selective higher-risk subgroups, and those targeting the individuals at the highest risk [3]. Prevention efforts which target selective subgroups may be effective at preventing both suicidal ideation and completed suicides in communities, according to recent research [3,4]. In order to proactively prevent suicide by targeting high-risk subgroups, these high-risk communities and their characteristics must be identified. This has been the subject of numerous studies as there has been extensive research on the individual and contextual level variables that attenuate or exacerbate suicide. Perhaps the most important of these are economic factors including unemployment, recessions, and poverty, all of which previous research has concluded are positively correlated with suicide rates [5,6,7,8,9].

The intent of this study is to develop a tool for predicting the risk of suicide using factors that contribute to suicidality. Counties serve as an agent of the state and unit of local government and provide vital services and resources to their citizens including schools, healthcare, infrastructure, parks, transportation, and public safety. Understanding the factors that play a role at the county level is valuable because it is the level of government that has the most direct impact on a citizen [10]. The National Institute of Mental Health (NIMH), as well as medical research centers including Mayo Clinic, instruct people to call emergency services if they believe that someone is in immediate danger of suicidal actions [11,12]. Police departments and Emergency Medical Services, both of which are most often organized and managed by county governments, are frequently the ones to intervene in a suicide crisis. Therefore, studying suicide at the county level may be the best way to address specific community needs and implement or improve suicide prevention plans.

Understanding suicide risk at the community level can provide valuable knowledge to proactively address rising suicide rates and create a framework for how to allocate resources to where they are most needed. While mental health resources are sporadic and increased risk will not inherently lead to more prevention efforts, it is nevertheless worth studying suicide at the county level because patterns in suicide differ across population subcultures and these differences are underexplored in research [13,14].

## 2. Suicidology

Suicidology, the study of suicide, is embedded within the larger constructs of psychology, sociology, anthropology, and most recently biology. Presented below is a review of the research on these four constructs and the variables that previous research has linked to suicide. Emphasis is given to those variables which link to the present study.

### 2.1. Psychology

Psychology primarily focuses on suicidality in individuals, and factors that influence suicidality at the individual level. Psychological theories of suicide consider varying personal traits and emotions that contribute to suicide, but social isolation is a common thread between these theories. The vulnerability model states that there are certain psychological characteristics that may make an individual more vulnerable to suicide [15]. While certain conditions can lead any individual to consider, attempt, or complete suicide, some individuals possess particular characteristics that predispose them to suicidality [15].

The Shame model, developed by Marvin Lansky, asserts that shame is the primary contributor to suicidality and describes shame as both the effect of failed social attachments and a hinderance to creating meaningful social bonds [16]. According to this model, shame contributes to suicidality more so than depression or guilt [15]. A study of suicide attempt survivors in Ghana identified shame as one of the most common reasons for suicide among men [17].

The Interpersonal Theory of Suicide, developed by Thomas Joiner, is one of the most widely studied and cited theories of suicide in the field of psychology. A meta-analysis published in 2019 identified 114 empirical studies including the theory, and Joiner’s book in which he outlines the theory boasts over five-thousand citations [18]. This theory puts social isolation and perceived burdensomeness at the center of suicidality in individuals. However, critics of the Interpersonal Theory of Suicide argue that the theory is not as universal as researchers claim because it considers suicide solely from a psychological perspective [18]. This weakness in Joiner’s theory reinforces that suicide is a complex problem that is molded not only by an individual, but also by their milieu, and must be studied as such [19].

While predicting such personal characteristics as social isolation or loneliness accurately at the county level is likely infeasible, it is possible to measure outside influences that, in combination with cultural values, are contributing factors to social isolation. In the United States and cultures with comparable values of self-reliance, financial instability is a source of shame among individuals, a result of the emphasis that America’s individualist culture places on achieving both financial stability and independence [17,20].

Numerous studies have supported the notion that suicide is associated with an individual’s financial status, but rather that association is positive or negative depends on both the population of interest and other factors at play [21,22,23]. These studies have spanned numerous nations across the globe but include considerably more Western countries [6,20,24,25]. Studies of survivors of suicide attempts in South Korea and Europe found financial stress to be one of the most commonly reported reasons for a suicide attempt for middle-aged individuals, while a similar study of female suicide attempt survivors in Cambodia found poverty to be one of four reasons reported for attempting suicide [22,26,27]. Quantitative analyses of suicide rates in Brazil, Germany, China, and other nations have concluded that economic deprivation is positively correlated with rates of death by suicide, while economic success is negatively correlated with rates of death by suicide [5,17,28,29].

Financial instability can contribute to an increase in suicide rates by increasing feelings of shame, humiliation, and inadequacy among people. Additionally, the self-reliant nature of American culture and the socially imposed goal of financial success can also increase feelings of shame and additional mental health problems among individuals who face financial dependence or instability [30]. For this reason, both measures of financial instability and the quality of mental health among individuals in a community were included in the model for predicting suicide risk. Variables used as indicators of mental health in a community were measures of alcohol and drug use, the size of the veteran population, and the average daily sunlight rate. Sunlight is a primary source of vitamin D, which has a positive impact on mood and mental health. Some previous studies have found positive associations between suicide and a colder climate or lack of sunlight [21,31,32,33]. Variables used to measure financial instability were the rates of poverty and home ownership and the median household income.

### 2.2. Sociology

Within sociology, suicidology studies consider how the norms and values within a society influence the occurrence of suicide. Sociological theories explaining suicide include the Theory of Anomie and Social Strain Theory, both of which consider social isolation as a significant factor [34]. Not only has social isolation been extensively studied as a suicide risk factor, but research has found that interpersonal trust is a crucial factor in protecting against suicide risk [24].

Sociological studies of suicide were made famous by Emile Durkheim’s Theory of Suicide, in which Durkheim defines the social and structural aspects of suicide [35]. The Theory of Suicide argues that social integration and adhesion to social norms protect against suicide, putting social isolation at the core of suicidality in the same way that psychology does [35,36,37]. Individuals who do not establish social ties and are not included within a social structure such as a family or religious group, and who do not adopt the social order created by norms and values, experience anomie. Anomie, defined as dysregulation, normlessness, and instability caused by a lack of socially established standards and social integration, leads to isolation, feelings of worthlessness, depression, and eventually to suicide [36].

Social Strain Theory was developed by Robert K. Merton from Emile Durkheim’s Theory of Anomie. The argument of Social Strain Theory is that every society has a defined system of norms and values, which includes goals that people within that society should strive to reach. In addition, included within the system of norms and values are the socially acceptable ways of reaching those goals [38]. Social strain emerges when a member of the society accepts these goals and aspires to reach them but is hindered from reaching them through the socially defined and accepted methods. There is a gap between one’s goal and the means, abilities, or resources they possess, which results in that person experiencing social strain and isolation. This strain creates an anomic environment within a person, which can put a person at risk for suicide [38].

Social isolation is the underlying common thread between sociological theories of suicide, as it is in psychological theories of suicide [20,39]. In the United States, there is a socially constructed and shared goal and expectation of independence that results from the individualistic culture. As a result, poverty, persistent unemployment, and downward social mobility can contribute to feeling disconnected from society, while opportunity, upward social mobility, and independence increase social integration [9,40].

One study found lack of employment to be one of the primary sources of anomie among males in the United States and cited the association between one’s work and their contribution to society to be a possible explanation [40]. A recent study concluded that increased income inequality had health and social consequences including chronic stress, depression, and shame and that these occurred mostly in low-income individuals [41]. Previous studies have also found that social isolation results from a lack of opportunity, lack of resources, and high levels of inequality, all of which act as barriers to social integration [9,28,41]. In summary, through employment, providing for oneself and one’s family, and upward social mobility, one is adhering to the socially constructed goal of self-reliance, and is integrated into their society. The inability to reach this shared goal pushes one to the peripheral of society and increases social isolation [42]. Variables measuring economic opportunity and financial stability, including unemployment rate, poverty, median household income, and the rate of home ownership, which economic studies have indicated is a means to build wealth and stability, were tested in the analysis [43]. Variables measuring individuals’ access to opportunity and potential for social mobility, including high school and college graduation rates, income inequality, rate of household internet access, and urbanicity (measured by population density) were tested as well.

Economic stability at the societal level has also been associated with suicide. Unemployment rates have been found to be positively correlated with suicide rates, while lack of health insurance, limited access to healthcare, high levels of social inequality, and inability to access education, housing, or other essential services have all been found to be risk factors for suicide in studies conducted in forty-four countries in total [5,28,29,44,45,46]. At the macro level, a study of fifteen countries found that there was a significant correlation between suicide rates and current economic conditions. Furthermore, the study found a significant positive relationship between the proportion of income that must be spent on essential expenses and the suicide rate. As it became harder for the people studied to cover their expenses with their income, the suicide rate increased [47]. These findings also may be partially explained by the correlation between financial instability and mental health problems. In the United States the prevalence of major depression significantly increased between 2005 and 2006, and between 2011 and 2012, both of which were times of economic recession when many people lost their jobs and homes, and economic strain was widespread [48]. A study in Canada, the United States, and the European Union concluded that the 6.5% increase in suicide in the EU and 4.8% increase in suicide in the U.S. between 2007 and 2009 lead to 10,000 more deaths from suicides than would have been expected under the economic conditions preceding 2007 [48]. A study that also considered the 2008 global economic recession found that suicide rates increased in men during this time period in all 54 countries considered [6]. A study in Italy that considered the same time period found an association between the global economic recession and a significant increase in suicide among men in the labor force [25]. As these studies spanned across multiple countries, the correlation between economic conditions and suicidality appears to hold constant for many societies, though future research should strive to include more non-Western nations in studies of economic conditions and suicide rates.

### 2.3. Anthropology

Within anthropology, the study of suicide considers the cultural implications of suicide, including how suicide is defined within a culture and what members of that culture believe happens after suicide completion [14]. Because even the very definition of suicide can vary between cultures, studies of suicide must include a cultural perspective to ensure that the information collected is interpreted in a manner that is relevant to the culture from which it came [49]. Even among groups of people from very similar cultures, there can be differences in perceptions of suicide. A study in the United States found that residents of Honor States, primarily southern states who equate self-worth and reputation, and residents of Dignity States, primarily northern states who see self-worth as being self-defined and not based on others’ perceptions of them, differ in their beliefs about suicide. Members of honor cultures reported more suicidality resulting from shame than members of dignity cultures [39].

Shame, like economic conditions, is associated with social isolation. Some psychologists have concluded that social connections protect against feelings of shame while social isolation puts one at higher risk for feelings of shame [50]. Another argument is that shame is a response to failing to reach the expectations imposed on individuals by their society [51]. It is worth noting that these two arguments for the source of shame are far from mutually exclusive, as evidenced by Merton’s studies which concluded that social isolation results from a failure to meet the goals created and shared by a society [9].

The anthropological perspective of suicidality emphasizes the importance of considering and studying the beliefs about suicide that vary between cultures and subcultures. Characteristics of a culture impact suicide rates by determining what situations are likely to lead suicide, such as social isolation has been shown to do in the United States. The United States scores highest of all ranked countries on the Individualism Index, developed to measure the importance that a nation places on individualism compared to collectivism [52,53]. Highly individualistic societies such as the United States value independence, self-reliance, and immediate family bonds, whereas collectivist societies place more emphasis on interpersonal relationships, individuals’ roles in groups, and a system of recidivism that both strengthens and relies on extended family bonds [52,54]. Furthermore, individualism in a society is positively correlated with shame and social withdrawal [55]. The extremely individualistic nature of American society creates an environment where social bonds are already looser and a strong emphasis on independence puts people at higher risk for feelings of shame, further solidifying the association between drivers of social isolation and suicide in the United States.

### 2.4. Modern Research: Neurobiology Model

Psychologists, sociologists, and anthropologists began to identify some of the factors of suicide before the end of the 19th century. Durkheim proposed the importance of social integration within members of a society, and the detrimental effects of social isolation in his 1897 book *Suicide*, while a study in Italy identified economic fluctuations as an important factor in 1881 [25,36,56,57]. Today, modern medical technology has prompted suicide research to expand into the fields of neurology and biology. The Neurobiology model evaluates molecular alterations, gene patterns and receptor signaling to link neurological and genetic abnormalities to suicidal behaviors and has dominated much of the recent research on suicide [3,58]. However, like socio-demographic or economic factors of suicide, neurology and genetics cannot be considered in isolation of social and environmental factors. For example, the research has indicated that serotonin dysregulation is a highly important factor in suicidality, but serotonin dysregulation is a result of genetics, environment, or both [3,58]. While the neurobiological model can provide extensive insight in suicidality in individuals, limited data and make it difficult to rely on the neurobiological model to create a framework by which to identify population subgroups at high risk for suicide.

## 3. Present Study

This study seeks to determine the quantitative variables that can be used to predict suicide at the county level. The guiding research question is: using the research on suicide, are there variables studied at the community level that are linked to suicide and are measurable using quantitative, demographic data that are already collected and updated? This study is advantageous because it is one of the few to comprehensively predict suicide at the county level and it is efficient since the necessary data are already available.

All variables included data at the county level. Analyzing suicide at the county level, rather than the state or national level, retains more of the variation in suicide rates, which can expose important trends in deaths from suicide. At the state level, the rate of suicide ranges from 5.1 to 27.2 deaths from suicide per 100,000 people. At the county level, however, the rates range from less than 4.7 deaths by suicide per 100,000 people to 49.1 deaths per 100,000 people [1]. Preserving variation allows for better identification of trends and patterns in the data and may reveal opportunities for insight to be gained from outlier analysis.

This study is unique in that it presents a comprehensive model for classifying counties based on suicide risk and incorporates a multi-disciplinary theoretical explanation for the variation in suicide risk between counties. To the best of the researchers’ knowledge, this study is the first to use cluster analysis as a tool for identifying high risk counties where suicide prevention efforts should be focused.

## 4. Methods

The predictor variables were grouped into four categories. Financial variables were poverty, rate of home ownership, and median household income. Variables for inequality and resource accessibility were unemployment rate, income inequality (the difference between the first and third income quartiles), urbanicity (population density), and high school and college graduation rates. Variables measuring the risk for mental health problems were the rates of drug and alcohol related deaths, the prevalence of mental health practitioners in the county compared to the national average, the percent of the population that are veterans, the average daily amount of sunlight, net population change, and the prevalence of household internet connections. Data for the county population, the average daily amount of solar radiation (sunlight) received, number of deaths from suicide, and the prevalence of drug and alcohol related deaths (two separate variables) were collected by the CDC and provided by the CDC Wide-ranging Online Data for Epidemiologic Research (CDC WONDER) [59]. The count of deaths from suicide for each county were compiled using official death reports filed by each county. The number of deaths from drug related causes and from alcohol related causes also came from official death reports filed in each county. Data were used from the years 2000, 2005, 2010 and 2015 for each variable.

Data on the percent of people living in poverty and the median household income at the county level were collected by the United States Census Bureau American Community Survey (ACS) and Decennial Census data archives for the years 2000, 2005, 2010, and 2015 [60,61]. Data for the population each year, as well as the percent of the population living in poverty were collected using the decennial census in 2000 and 2010 and the American Community Survey in 2005 and 2015 [60,61]. Data on the county level unemployment rate for the years 2000, 2005, 2010, and 2015, and the prevalence of mental health practitioners in each county, were provided by the Bureau of Labor Statistics through the Current Population Survey and the Quarterly Census of Employment and Wages (QCEW) [62]. Lastly, data on the prevalence of households in each county with internet access for the years 2000, 2005, 2010, and 2015 were collected and made publicly available by the Federal Communications Commission (FCC), which uses both Information Technology services as well as data submitted by internet providers to determine internet connection rates [63].

Using SAS 9.4, these datasets were merged to form one dataset with the following variables: number of deaths from suicide, year, population, sunlight, rate of household internet connections, unemployment, number of drug related deaths, number of alcohol related deaths, percent of people living in poverty, median household income, prevalence of mental health practitioners, number of veterans, population density, population change, the rate of home ownership, and income inequality. Each observation was a county in the United States, identified by the county’s Federal Information Processing Standard (FIPS) code. Each county had up to four observations, one for each time point of the study: 2000, 2005, 2010, 2015. Counties for which the number of suicide deaths was missing were removed from the dataset. 3252 observations remained in the final dataset. For observations that had a missing value for one of the variables, the missing value was replaced with the median value. The distribution of all variables except the rate of home ownership was skewed right, and so a log transformation was done on each skewed variable. The final step in the data management stage was to convert counts into rates so that differences in county population were accounted for when calculating the prevalence of a particular variable. The number of deaths from suicide, drug related deaths, and alcohol related deaths were each divided by the total county population for the respective year, and then the decimal value multiplied by 1000 to result in a number representing the number of deaths per 1000 county residents.

### Data Analysis Approach

Linear regression analysis using SAS was conducted first to check for multicollinearity and to identify significant predictor variables. Multiple linear regression, also using SAS, was then completed to model the importance of each contributing factor. Following multiple linear regression, SAS was used to create a longitudinal model to determine where changes in the predictor variables were significantly correlated with change in the rate of death by suicide.

The final stage of analysis consisted of creating multiple clustering algorithms in order to classify counties by predicted suicide risk based on the independent variables considered. Four clustering methods were used to create models: multi-level perceptron classifier, K-means, K-medoid, and hierarchical clustering methods. The multi-level perceptron classifier is an artificial neural network method that uses an input layer, an output layer, and multiple hidden layers to learn and classify data that may be non-linear. The K-means and the K-medoids clustering methods are similar in that both minimize the distance between the points in a cluster, but in the K-means method the center of each cluster is a mean value, whereas in the K-medoids method the clusters are centered around an actual data point and not a mean [64]. The method of hierarchical clustering used in this analysis splits the data into two groups, and then continues to split those groups into smaller clusters [64]. The silhouette width, a measure of the average distance between each cluster, was used to determine the efficacy of the K-medoids clustering algorithm, while the efficacy of the other three methods was measured using the proportion of data points that were consistently and accurately classified based on current suicide rates using only independent variables. The goal of a clustering algorithm is to be able to classify counties based only on a few independent variables so that counties for which data are limited can be classified by suicide risk, and so that areas of high or increasing suicide risk can be identified preemptively, even in places where suicide data are unavailable. It is in these counties where this algorithm may perhaps be most useful.

Building a model to predict suicide rates based on quantitative factors allows for a more proactive approach to suicide prevention by sooner identifying characteristics of communities that are at risk for increases in suicide. Suicide prevention initiatives can be aimed at the factors contributing to the increases in suicide rate in communities and can be tailored to individual communities. Classifying counties based on suicide risk can provide a framework to allocate resources to communities in need. A classification model can also allow similar counties to be investigated further to identify additional sociological or cultural factors contributing to or protecting against suicidality.

It is necessary to emphasize here that the data evaluated were not nested. Aggregate information was provided for each of the 1000 counties. Information about individuals or neighborhoods within each county was not tested.

## 5. Results

Multiple regression analysis resulted in a model with ten significant predictor variables and an adjusted R^2^ value of 0.63. While population density, prevalence of drug related deaths, and veteran population had small parameter estimates in comparison to others predictor variables, partial F-tests indicated that the model was significantly compromised when these variables were removed from the model. Therefore, all ten significant variables were retained in the final model (Table 1).

Longitudinal analysis revealed that changes in drug and alcohol related death rates were most strongly correlated with changes in rates of suicide over the fifteen-year span of the data, with drug abuse rates having the strongest effect on rates of suicide (Table 2).

**Figure 1:**
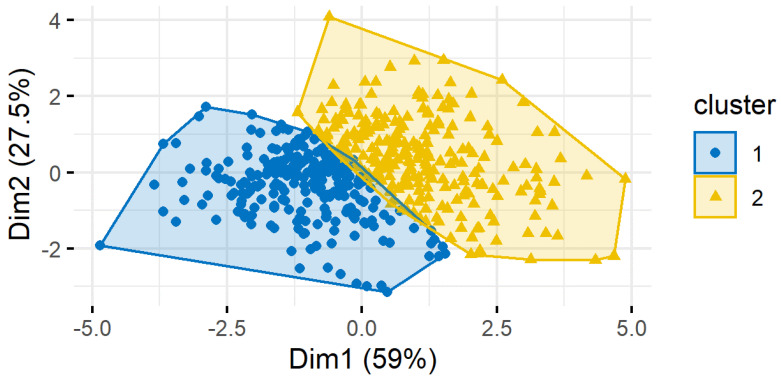
K−Medoid Method Clustering with k = 2 clusters.

**Figure 2:**
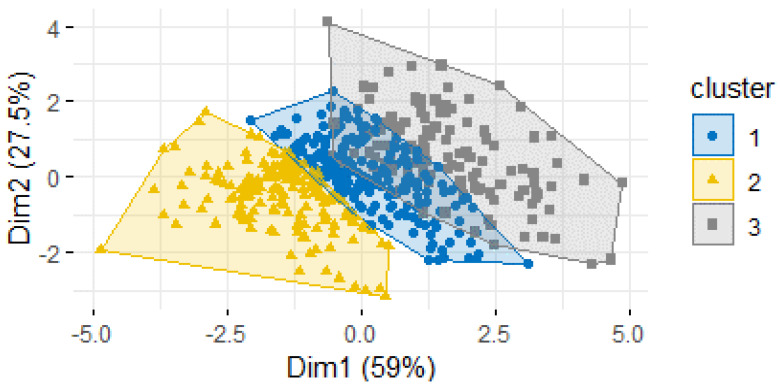
K−Medoid Method Clustering with k = 3 clusters.

Lastly, multiple clustering algorithms were created to classify counties into groups based on suicide risk. Variables included were median household income, poverty, income inequality, unemployment, home ownership, high school and college graduation rates, net population change, drug and alcohol related death rates, prevalence of mental health practitioners, population density, household internet connection rate, and daily sunlight. Calculating silhouette width, a measure of similarity within clusters and difference between clusters, was used to measure consistency of the clustering algorithm. The clustering algorithms with the best measure of silhouette width included the following predictors: median household income, alcohol and drug related death rates, and poverty rate, and the K-medoids method created the most successful clustering algorithm. The K-medoids algorithm resulted in the most counties being assigned to the cluster corresponding to their current suicide rate based only on five independent variables and had the largest silhouette width. Two (Figure 1) or three (Figure 2) clusters resulted in the most accurate classification of counties. Specifically, results showed that counties with a higher rate of death by suicide also have a higher rate of drug and alcohol related deaths, higher poverty rate, and lower median household income. Clustering the counties based on similar demographic features that may put them at a higher suicide risk is a method for quickly identifying counties where resources may be needed most.

## 6. Discussion

External risk factors are under-researched compared to internal risk factors. This study fills a gap in the research on suicide by comprehensively studying the external and county level risk factors that are linked to suicide. Additionally studied were the variables linked to changes in suicide rate over time, which showed that the suicide rate in the average county changes with the rate of substance abuse and internet usage.

Substance abuse measures proved to be a highly important when building a predictive model of suicide risk. The importance of substance abuse may be explained by Zhang’s Strain Theory of Suicide, which builds off of the Theories of Anomie and Social Strain developed by Durkheim and Merton [65]. Durkheim and Merton theorized that the inability to integrate into society leads to anomie and social strain and puts the individual at risk of suicide [36,38]. Zhang further theorized that when mental illness is coupled with social strain and deficient coping, then the risk of suicide is significantly increased [65]. Substance abuse represents one form of maladaptive coping with social strain.

While social isolation, social strain, or shame may explain the relationship between poverty or financial instability and suicide, and economic explanation may also be at play. Increased spending on health and wellbeing and social welfare is correlated with lower suicide rates in the United States. However, counties with high levels of poverty or financial instability among residents are less likely to have the resources for robust public health and welfare programs. They also have a larger portion of the population among which to distribute the resources that are available [21].

Economic conditions, resources, and opportunity make up the third group of factors in the models and include education, inequality, urbanicity, and internet access. One possible explanation for the relationship between population density and suicide is access to social welfare resources, since social welfare spending is known to be positively correlated with suicide rates. Analysis of data from three major US cities showed a significant difference in access to social welfare resources for impoverished individuals just between urban and suburban neighborhoods [66]. This difference is likely larger between rural and urban communities.

Income inequality had a negative relationship with suicide rate which may be explained by the method of measuring income inequality. Income inequality was measured by subtracting the 25th percentile median household income value from the 75th percentile value, creating a value representing the range between lower income and higher income residents in the county. Therefore, counties with high income inequality may also have a higher 75th percentile MHI value, and therefore, more residents with greater access to resources, health care, etc. More research is necessary to determine the impact of income inequality on suicide in the United States.

The relationship between internet and suicide is a newer research area that is still in great need of further analysis. This topic recently made headlines after The Washington Post published an exposé alleging that Facebook had evidence that Instagram was contributing to worsening mental health conditions in teenage girls but did not inform the public. While they could not conclude that Instagram was the cause of declining mental health in teenage girls, the United States Senate subsequently released a report highlighting the correlations between internet usage, specifically social media, and rising mental illness and suicide rates in teenagers [67]. One explanation to consider is whether media access is a latent variable that connects internet access and suicide. Studies have indicated that how suicide and suicidal behavior is portrayed by media outlets may increase suicidality in the greater population [68]. Internet access may also allow vulnerable individuals to be exposed to material that can increase suicidal behavior, either through directly encouraging suicide or by providing information on methods of suicide [68]. There is an urgent need for increased research in this area.

The COVID-19 pandemic has aggravated several of the risk factors in suicide. Economic decline and uncertainty have resulted in many people experiencing a significant decline in socio-economic status and financial stability. As more aspects of people’s lives have moved to online platforms, including education, work, healthcare, and relationships with family and friends, the amount of time spent online has increased for many people. Pandemic conditions also resulted in increased social isolation as people were prevented from visiting family or friends, worked or attended school from home, and were unable to participate in religious traditions, sports, or other social groups [69,70,71,72]. One study found that suicide related online searches increased considerably during the pandemic [69]. A combination of increased social isolation and social strain lead to significant increases in mental health concerns during the pandemic [70]. The increase in suicide risk factors resulting from the current social and economic conditions make identifying the population subgroups at the highest risk a priority in order to mitigate the potential increase in suicidal behavior.

Given the recent interest in the role of neurobiology on suicide, this area of research should not be studied in isolation, but rather should be integrated with the work on environmental, psychological, and social factors that impact suicide in order to provide a comprehensive understanding of suicidal behavior. A longitudinal and developmental perspective is ideal for this area of research. Every county included in this study that had low rates of suicide will have individuals who are still at high-risk of suicide. The scope of this study does not extend to the psychiatric or neurobiological traits of individuals that impact the risk of suicide at the individual level. The limitations of this study are that no individual data are used, and so it is impossible to ascertain how an individual at high-risk for suicide may be impacted by residing in a low-risk county, and vice versa. This study may be further strengthened by the use of nested county data which would allow for the study of different neighborhoods within counties.

## 7. Conclusions

In the final models, ten factors were found to be important in predicting the county level suicide rate: median household income, poverty, income inequality, home ownership, high school graduation rate, population density, alcohol related deaths, drug related deaths, sunlight, and the percentage of the population that are veterans. The longitudinal analysis results further revealed the importance of drug and alcohol related deaths in predicting suicide rates, as well as the role that increasing internet access plays. The correlation between substance use in a community and the suicide rate opens a door to additional sources of information that may contribute to the knowledge of suicide risk in a community, since substance abuse information is collected by both law enforcement and health care agencies.

Analysis in this study indicates that factors measuring financial stability (median household income, home ownership, and poverty), economic prospect (income inequality and high school graduation rates), and mental health quality (drug and alcohol related death rates, daily sunlight, and the prevalence of veterans) can be used to predict suicide risk in counties. The availability of data on these demographic variables makes the multiple regression model and the clustering algorithm for predicting suicide more powerful, since they can predict an estimate of suicide risk for counties where suicide data are unavailable.

Addressing the problems of financial insecurity and poor economic conditions in a community is one important step toward lowering suicide risk. However, the importance of the variables measuring the quality of mental health in the models, including drug and alcohol related deaths, sunlight, and the veteran population, indicates that increasing economic prospect and financial independence alone would be neglecting a key aspect of suicide prevention. In addition, highlighted is the link between financial stability, social class, and mental health in the United States. American culture places high importance on achieving self-reliance and avoiding downward social mobility, which is supported by the interwoven relationships between financial and economic conditions, shame, and suicide behaviors. Efforts to improve mental health and prevent suicide in communities with limited economic growth and widespread poverty are critical in preventing suicide, but must also address ways to improve mental health among people experiencing these conditions.

Eliminating poverty, unemployment, and periods of poor economic conditions has thus far been infeasible, and so focus must shift to treating the mental health problems that are exacerbated by these conditions, especially in the aftermath of the COVID-19 pandemic. This study provides a framework for identifying where research and resources should be focused in order to have the largest impact on the rate of death by suicide in communities across the United States, as well as a model for identifying where people are at the highest risk for suicide.

## Figures and Tables

**Table 1:** Multiple Regression Model Results.

Independent Variable	Parameter Estimate	*p*-Value
Median Household Income	0.23635	0.0001
Poverty	0.10560	0.0001
Income Inequality	−0.29825	0.0001
Home ownership	0.21399	0.0001
High school graduation rate	0.46011	0.0001
Population Density	−0.03714	0.0001
Alcohol related deaths	0.12906	0.0001
Drug related deaths	0.04395	0.0001
Sunlight	0.17428	0.0001
Veteran population	0.04593	0.0001

**Table 2:** Longitudinal Analysis Model Results.

Effect	Parameter Estimate	Standard Error	Degrees of Freedom	t-Value	*p*-Value
Intercept	0.03107	0.00762	1034	4.08	0.0001
Rate of Alcohol related deaths	0.9951	0.06652	766	14.96	0.0001
Rate of drug related deaths	1.0478	0.06838	766	15.32	0.0001
Household internet connections	0.00007	0.00001	766	6.72	0.0001

## Data Availability

Publicly available datasets were analyzed in this study. This data can be found here: CDC WONDER: Available online: https://wonder.cdc.gov/ (accessed on 19 August 2020); United States Bureau of Labor Statistics Quarterly Census of Employment and Wages: Available online: https://www.bls.gov/cew/ (accessed on 4 September 2020); United States Census Bureau: Available online: https://data.census.gov/cedsci/ (accessed on 24 August 2020); United States Federal Communications Commission: Available online: https://www.fcc.gov (accessed on 4 October 2020).

## References

[1] Center for Disease Control and Prevention (CDC) WISQARS-Web-Based Injury Statistics Query and Reporting System. https://www.cdc.gov/injury/wisqars/index.html.

[2] Deisenhammer E.A., Ing C.-M., Strauss R., Kemmler G., Hinterhuber H., Weiss E.M. (2008). The Duration of the Suicidal Process. J. Clin. Psychiatry.

[3] Turecki G., Brent D., Gunnell D., O’Connor R., Oquendo M., Pirkis J., Stanley B. (2019). Suicide and suicide risk. Nat. Rev. Dis. Primers.

[4] Saewyc E.M., Konishi C., Rose H.A., Homma Y. (2014). School-based strategies to reduce suicidal ideation, suicide attempts, and discrimination among sexual minority and heterosexual adolescents in western Canada. Int. J. Child Youth Fam. Stud..

[5] Alarcão A.C., Dell’Agnolo C.M., Vissoci J.R., Carvalho E.C., Staton C.A., de Andrade L., Fontes K.B., Pelloso S.M., Nievola J.C., Carvalho M.D. (2020). Suicide mortality among youth in southern Brazil: A spatiotemporal evaluation of socioeconomic vulnerability. Braz. J. Psychiatry.

[6] Chang S.-S., Stuckler D., Yip P., Gunnell D. (2013). Impact of 2008 global economic crisis on suicide: Time trend study in 54 countries. BMJ Br. Med. J..

[7] Jaen-Varas D., Mari J.J., Asevedo E., Borschmann R., Diniz E., Ziebold C., Gadelha A. (2019). The association between adolescent suicide rates and socioeconomic indicators in Brazil: A 10-year retrospective ecological study. Braz. J. Psychiatry.

[8] Korhonen M., Puhakka M., Viren M. (2017). Economic hardship and suicides. Int. J. Soc. Econ..

[9] Kubrin C.E., Wadsworth T., DiPietro S. (2006). Deindustrialization, Disadvantage and Suicide among Young Black Males. Soc. Forces.

[10] Advisory Commission on Intergovernmental Relations (1979). Citizen Participation in the American Federal System.

[11] National Institute of Mental Health (NIMH) Suicide Prevention. https://www.nimh.nih.gov/health/topics/suicide-prevention/.

[12] Mayo Clinic Suicide: What to Do When Someone Is Suicidal. https://www.mayoclinic.org/diseases-conditions/suicide/in-depth/suicide/art-20044707.

[13] Choi M., Lee Y. (2020). Regional Variation of Suicide Mortality in South Korea. Int. J. Environ. Res. Public Health.

[14] Lester D. (2012). The Cultural Meaning of Suicide: What does that mean?. OMEGA-J. Death Dying.

[15] Ronningstam E., Weinberg I., Maltsberger J., Wasserman D., Wasserman C. (2009). Psychoanalytic theories of suicide–Historical overview and empirical evidence. Oxford Textbook of Suicidology and Suicide Prevention.

[16] Lansky M. (1991). Shame and the Problem of Suicide: A Family Systems Perspective. Br. J. Psychother..

[17] Akotia C.S., Knizek B.L., Hjelmeland H., Kinyanda E., Osafo J. (2018). Reasons for attempting suicide: An exploratory study in Ghana. Transcult. Psychiatry.

[18] Hjelmeland H., Knizek B.L. (2019). The emperor’s new clothes? A critical look at the interpersonal theory of suicide. Death Stud..

[19] Boldt M. (1988). The meaning of suicide: Implications for research. Crisis.

[20] Cooper V., Whyte D. (2017). The Violence of Austerity.

[21] Minoiu C., Andrés A.R. (2008). The effect of public spending on suicide: Evidence from U.S. state data. J. Socio-Econ..

[22] Seponski D.M., Somo C.M., Kao S., Lahar C.J., Khann S., Schunert T. (2019). Family, Health, and Poverty Factors Impacting Suicide Attempts in Cambodian Women. Crisis.

[23] Stack S., Wasserman I. (2007). Economic Strain and Suicide Risk: A Qualitative Analysis. Suicide Life-Threatening Behav..

[24] Economou M., Madianos M., Peppou L., Theleritis C., Patelakis A., Stefanis C. (2013). Suicidal ideation and reported suicide attempts in Greece during the economic crisis. World Psychiatry.

[25] Pompili M., Vichi M., Innamorati M., Lester D., Yang B., De Leo D., Girardi P. (2014). Suicide in Italy during a time of economic recession: Some recent data related to age and gender based on a nationwide register study. Health Soc. Care Community.

[26] Burón P., Jimenez-Trevino L., Saiz P.A., García-Portilla M.P., Corcoran P., Carli V., Fekete S., Hadlaczky G., Hegerl U., Michel K. (2016). Reasons for Attempted Suicide in Europe: Prevalence, Associated Factors, and Risk of Repetition. Arch. Suicide Res..

[27] Kim S.H., Kim H.J., Oh S.H., Cha K. (2020). Analysis of attempted suicide episodes presenting to the emergency department: Comparison of young, middle aged and older people. Int. J. Ment. Health Syst..

[28] Helbich M., Blüml V., De Jong T., Plener P.L., Kwan M.-P., Kapusta N.D. (2017). Urban–rural inequalities in suicide mortality: A comparison of urbanicity indicators. Int. J. Health Geogr..

[29] Hsu C.-Y., Chang S.-S., Lee E.S., Yip P.S. (2015). Geography of suicide in Hong Kong: Spatial patterning, and socioeconomic correlates and inequalities. Soc. Sci. Med..

[30] Hughes M., Kiecolt K.J., Keith V.M. (2013). How Racial Identity Moderates the Impact of Financial Stress on Mental Health among African Americans. Soc. Ment. Health.

[31] Dunham K.L. (1992). Seasonal Affective Disorder: Light Makes Right. Am. J. Nurs..

[32] Penckofer S., Kouba J., Byrn M., Ferrans C.E. (2010). Vitamin D and Depression: Where is all the Sunshine?. Issues Ment. Health Nurs..

[33] Thorson J., Kasworm C. (1984). Sunshine and suicide: Possible influences of climate on behavior. Death Educ..

[34] Gunn J., Lester D. (2014). Theories of Suicide: Past, Present and Future.

[35] Pickering W., Walford G. (2000). Durkheim’s Suicide: A Century of Research and Debate.

[36] Durkheim E., Simpson G. (1967). Suicide: A Study in Sociology.

[37] Stanley I.H., Hom M.A., Rogers M., Hagan C.R., Joiner T.E. (2015). Understanding suicide among older adults: A review of psychological and sociological theories of suicide. Aging Ment. Health.

[38] Kivisto P. (2011). Key Ideas in Sociology.

[39] Crowder M.K., Kemmelmeier M. (2017). Cultural Differences in Shame and Guilt as Understandable Reasons for Suicide. Psychol. Rep..

[40] Powell E.H. (1958). Occupation, Status, and Suicide: Toward a Redefinition of Anomie. Am. Sociol. Rev..

[41] Polacko M. (2021). Causes and Consequences of Income Inequality–An Overview. Stat. Politics Policy.

[42] Adler P., Du Gay P., Morgan G., Reed M. (2014). The Oxford Handbook of Sociology, Social Theory, and Organization Studies: Contemporary Currents.

[43] Goodman L.S., Mayer C. (2018). Homeownership and the American Dream. J. Econ. Perspect..

[44] Machado D.B., Rasella D., Dos Santos D.N. (2015). Impact of Income Inequality and Other Social Determinants on Suicide Rate in Brazil. PLoS ONE.

[45] Johnson K.F., Brookover D.L. (2020). Counselors’ Role in Decreasing Suicide in Mental Health Professional Shortage Areas in the United States. J. Ment. Health Couns..

[46] Liu X., Huang Y., Liu Y. (2018). Prevalence, distribution, and associated factors of suicide attempts in young adolescents: School-based data from 40 low-income and middle-income countries. PLoS ONE.

[47] Heydari A., Teymoori A., Nasiri H. (2014). The Effect of Parent and Peer Attachment on Suicidality: The Mediation Effect of Self-Control and Anomie. Community Ment. Health J..

[48] O’Hara M., Cooper V., Whyte D. (2017). Mental Health and Suicide. The Violence of Austerity.

[49] Abrutyn S., Mueller A.S. (2018). Toward a Cultural-Structural Theory of Suicide: Examining Excessive Regulation and Its Discontents. Sociol. Theory.

[50] Sznycer D., Takemura K., Delton A.W., Sato K., Robertson T., Cosmides L., Tooby J. (2012). Cross-Cultural Differences and Similarities in Proneness to Shame: An Adaptationist and Ecological Approach. Evol. Psychol. Int. J. Evol. Approaches Psychol. Behav..

[51] Maibom H. (2010). The Descent of Shame. Philos. Phenomenol. Res..

[52] Hofstede G. (2011). Dimensionalizing Cultures: The Hofstede Model in context. Online Read. Psychol. Cult..

[53] Braje I.N., Klindžić M., Galetić L. (2019). The role of individual variable pay in a collectivistic culture society: An evaluation. Econ. Res. -Ekon. Istraživanja.

[54] Minkov M., Dutt P., Schachner M., Morales O., Sanchez C., Jandosova J., Khassenbekov Y., Mudd B. (2017). A revision of Hofstede’s individualism-collectivism dimension: A new national index from a 56-country study. Cross Cult. Strateg. Manag..

[55] Young I.F., Razavi P., Cohen T.R., Yang Q., Alabèrnia-Segura M., Sullivan D. (2021). A multidimensional approach to the relationship between individualism-collectivism and guilt and shame. Emotion.

[56] Milner A., McClure R., De Leo D. (2012). Socio-economic determinants of suicide: An ecological analysis of 35 countries. Soc. Psychiatry Psychiatr. Epidemiol..

[57] Brancaccio M. (2013). “The Fatal Tendency of Civilized Society”: Enrico Morselli’s Suicide, Moral Statistics, and Positivism in Italy. J. Soc. Hist..

[58] Carballo J.J., Akamnonu C.P., Oquendo M.A. (2008). Neurobiology of Suicidal Behavior: An Integration of Biological and Clinical Findings. Arch. Suicide Res..

[59] Center for Disease Control and Prevention (CDC) CDC Wonder. https://wonder.cdc.gov/.

[60] United States Census Bureau (2020). American Community Survey. https://data.census.gov/cedsci/.

[61] United States Census Bureau (2020). Decennial Census. https://data.census.gov/cedsci/.

[62] United States Bureau of Labor Statistics (2020). Quarterly Census of Employment and Wages. https://www.bls.gov/cew/.

[63] United States Federal Communications Commission (FCC) Local Telephone Competition and Broadband Reporting. https://www.fcc.gov/licensing.

[64] Johnson R., Wichern D. (2007). Applied Multivariate Statistical Analysis: Clustering, Distance Methods, and Ordination.

[65] Zhang J. (2019). The strain theory of suicide. J. Pac. Rim Psychol..

[66] Allard S. (2004). Access to Social Services: The Changing Urban Geography of Poverty and Service Provision.

[67] Sheffield R., Francois C., United States Congress Joint Economic Commission Is Instagram Causing Poorer Mental Health among Teen Girls?. https://www.jec.senate.gov/public/index.cfm/republicans/2021/12/is-instagram-causing-poorer-mental-health-among-teen-girls.

[68] Hawton K., van Heeringen K. (2009). Suicide. Lancet.

[69] Costanza A., Amerio A., Aguglia A., Serafini G., Amore M., Macchiarulo E., Branca F., Merli R. (2021). From “The Interpersonal Theory of Suicide” to “The Interpersonal Trust”: An unexpected and effective resource to mitigate economic crisis-related suicide risk in times of COVID-19?. Acta Biomed..

[70] Nelson B.W., Pettitt A., Flannery J.E., Allen N.B. (2020). Rapid assessment of psychological and epidemiological correlates of COVID-19 concern, financial strain, and health-related behavior change in a large online sample. PLoS ONE.

[71] Wei X., Li L., Zhang F. (2021). The impact of the COVID-19 pandemic on socio-economic and sustainability. Environ. Sci. Pollut. Res..

[72] Wong C.W., Tsai A., Jonas J.B., Ohno-Matsui K., Chen J., Ang M., Ting D.S.W. (2021). Digital Screen Time during the COVID-19 Pandemic: Risk for a Further Myopia Boom?. Am. J. Ophthalmol..

